# Supplementary figures and images for: Assessing white matter plasticity in a randomized controlled trial of early literacy training in preschoolers
Source: PLoS One. 2025 Mar 19;20(3):e0309574. doi: 10.1371/journal.pone.0309574 (PMC11957728; doi:10.1371/journal.pone.0309574)

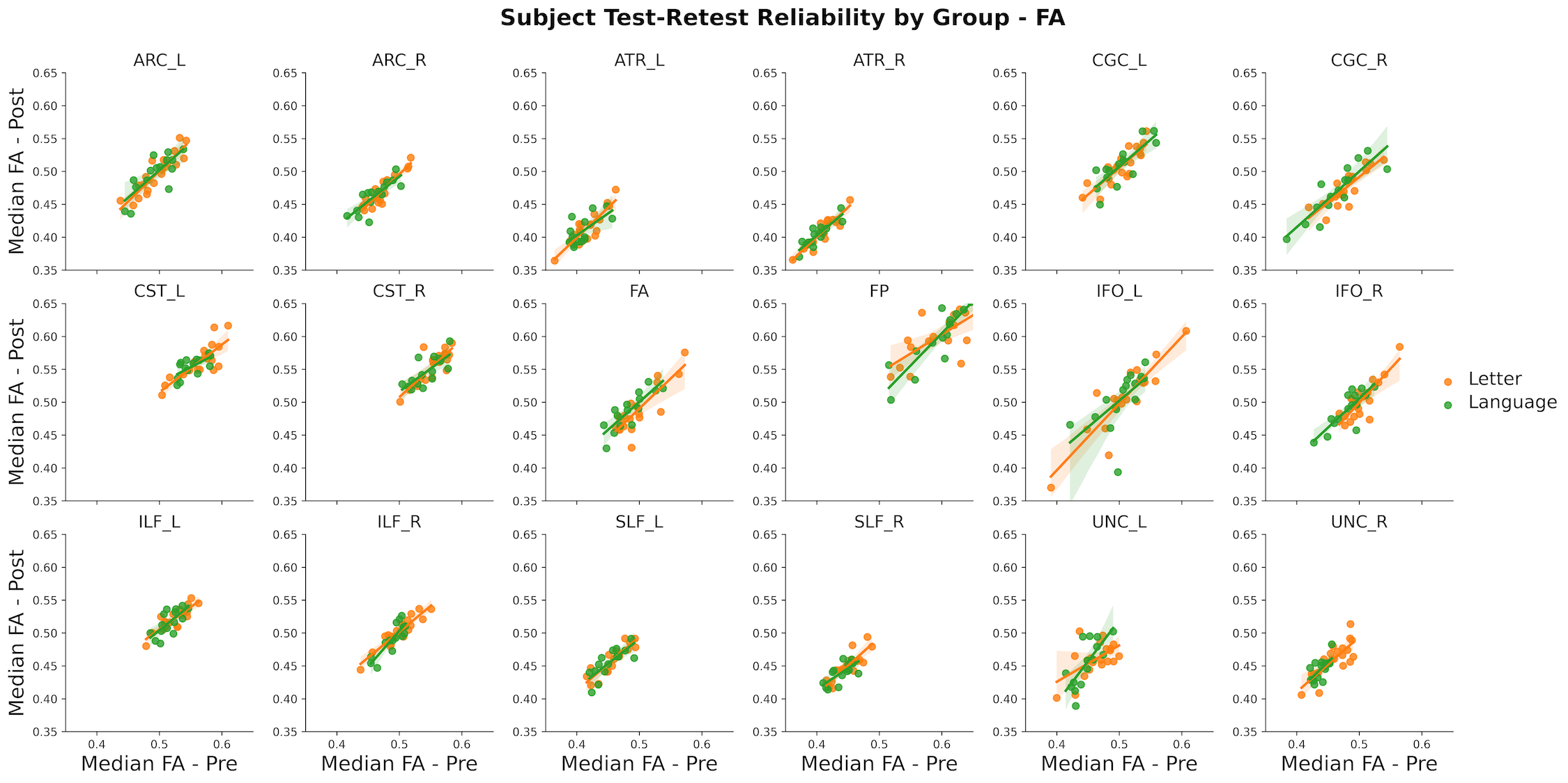

Supplement: S1 Fig — (TIFF) [file pone.0309574.s001.tiff]

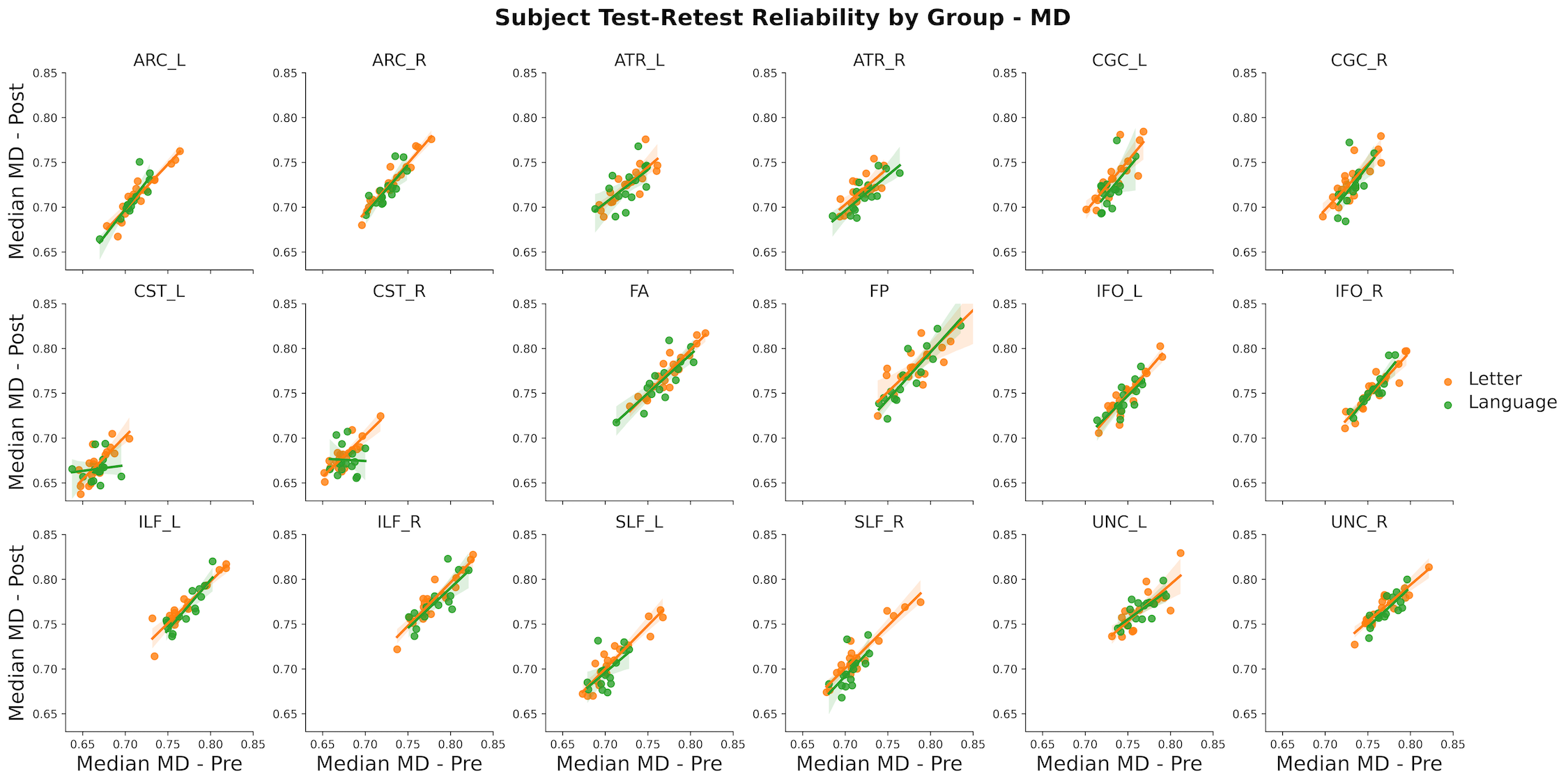

Supplement: S2 Fig — (TIFF) [file pone.0309574.s002.tiff]

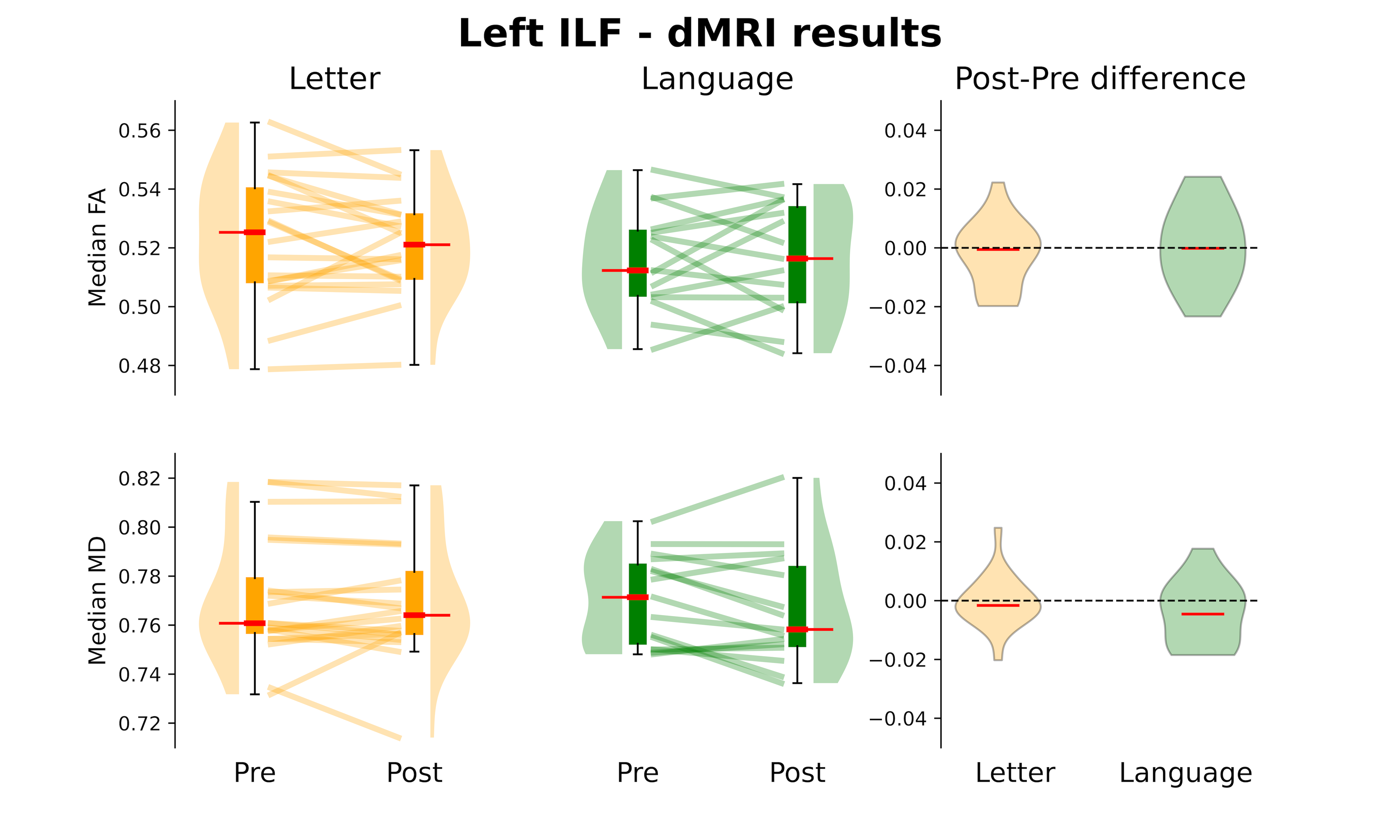

Supplement: S3 Fig — First two columns: structural changes of the left ILF are shown for the Letter and Language Training groups and each experimental session. The third column shows the distribution of FA and MD changes (i.e., difference between the individual profiles observed in the post and pre-training sessions) for each group. (TIFF) [file pone.0309574.s003.tiff]
