# Supplementary material for: Assessing white matter plasticity in a randomized controlled trial of early literacy training in preschoolers
Source: PLoS One. 2025 Mar 19;20(3):e0309574. doi: 10.1371/journal.pone.0309574 (PMC11957728; doi:10.1371/journal.pone.0309574)
Supplement: Attachment — Submitted filename: Response_to_reviewers_auresp_2.docx [file pone.0309574.s005.docx]

Reviewers' comments:

Have the authors made all data underlying the findings in their manuscript fully available?

The [PLOS Data policy](http://track.editorialmanager.com/CL0/http:%2F%2Fwww.plosone.org%2Fstatic%2Fpolicies.action%23sharing/1/010f0193b32e8d6a-bb6bbbe1-3230-45b0-ac24-ee7ba91f7adf-000000/7WMSLZ7NnyxUQiBfMmSoS4-hHnXSbTUDoEI_g44BIpQ=189) requires authors to make all data underlying the findings described in their manuscript fully available without restriction, with rare exception (please refer to the Data Availability Statement in the manuscript PDF file). The data should be provided as part of the manuscript or its supporting information, or deposited to a public repository. For example, in addition to summary statistics, the data points behind means, medians and variance measures should be available. If there are restrictions on publicly sharing data—e.g. participant privacy or use of data from a third party—those must be specified.

Reviewer #1: No

Reviewer #2: Yes

*We made sure that the openeuro link works. The full dataset is available here: PREK. OpenNeuro. [Dataset] doi: 10.18112/openneuro.ds005572.v1.0.0*

*Here is a review link where the dataset can be accessed anonymously:* [*https://openneuro.org/crn/reviewer/eyJhbGciOiJIUzI1NiIsInR5cCI6IkpXVCJ9.eyJzdWIiOiI[…]IjoxNzY4NDAxNzYyfQ.oaZHx1LDOZcFyh0qHt5b-lU1Y-pVZooV-HnYVuvXq5U*](https://openneuro.org/crn/reviewer/eyJhbGciOiJIUzI1NiIsInR5cCI6IkpXVCJ9.eyJzdWIiOiIwMDEzNjdhOS03Zjg4LTQ2OGYtOGUwNi03OTM1ZmIyNjBlOTAiLCJlbWFpbCI6InJldmlld2VyQG9wZW5uZXVyby5vcmciLCJwcm92aWRlciI6Ik9wZW5OZXVybyIsIm5hbWUiOiJBbm9ueW1vdXMgUmV2aWV3ZXIiLCJhZG1pbiI6ZmFsc2UsInNjb3BlcyI6WyJkYXRhc2V0OnJldmlld2VyIl0sImRhdGFzZXQiOiJkczAwNTU3MiIsImlhdCI6MTczNjg2NTc2MiwiZXhwIjoxNzY4NDAxNzYyfQ.oaZHx1LDOZcFyh0qHt5b-lU1Y-pVZooV-HnYVuvXq5U)

**Reviewer #1:**

The reviewers have carefully addressed all of my initial queries. I have several small suggestions pertaining to the revised version of the manuscript:

1. Data Availability: I attempted to access the data at doi: 10.18112/openneuro.ds005572.v1.0.0 and received the error “403: You do not have access to this page, you may need to sign in.” I was still unable to access the data after signing in with my ORCID.

*We made sure that the link works.*

*The full dataset is available here: PREK. OpenNeuro. [Dataset] doi: 10.18112/openneuro.ds005572.v1.0.0*

2. Table 2.
- Typo: Post MRI-camp time different (days)
- Please indicate whether Raw or Standard/Scaled scores are reported (also for the PPVT inclusion criteria, p.8 l. 147).

*We added these corrections to Table 2 and page 8 and we clarified that raw scores are reported.*

3. Methods
- Please cite software and packages used for statistical analysis.

*Statistical analyses were performed using python v3.11 and the following modules: scipy.stats v.1.15.0, statsmodels v0.14.4. We added this information to pages 14 and 17.*

4. Thank you for providing additional clarity regarding the reliability analyses. The inclusion of scan-rescan reliability analysis conducted over the two experimental sessions seems counterintuitive given the hypothesis that the DTI metrics would change rapidly with reading training. In other words, if the results had shown significant training-related changes in DTI measures, the reliability tests would not have shown strong scan-rescan reliability. I suggest further justification and discussion of the reliability analysis, or restructuring to include the reliability analysis in the supplementary materials to show the stability of the DTI metrics over time, rather than as a preliminary analysis. Otherwise, please clarify if I am misunderstanding the implementation of the reliability analysis in this study.

*High scan-rescan reliability does not necessarily exclude the presence of training-induced DTI changes. This is because Pearson correlation mainly checks if the overall relationship among participants (in the case of subject reliability) or tract nodes (in the case of profile reliability) is constant between the two experimental sessions but it’s not sensitive to changes in the scale of the variables. For instance, we did not expect that reading training would drastically change the relationships across participants between the two sessions. Instead we expected a general increase of FA while maintaining the overall between-subject distances constant. The presence of a shift in FA values between sessions is still compatible with high subject reliability, as Pearson correlations are invariant to changes in scale. We added a clarification on this point to page 18.*

5. Results
- Please clarify the direction of change in left ILF FA that is potentially associated with Alphabet Knowledge improvement. The statement that “BFs signaled a moderate support for the presence of a link between Alphabet knowledge improvement and an FA reduction of the left ILF (BF>3).” (P. 23, lines 392-394) seems to indicate that children who improved more on Alphabet knowledge showed a decrease in FA of the left ILF over time, but this does not seem to be consistent with the explanation in the text or the response to Reviewer 2, in which it seems that the negative correlation between Alphabet Knowledge change and FA change indicates that children with greater Alphabet Knowledge improvement showed smaller increases in FA. It would be helpful to include Figure S3 in the main text to support interpretation of these results.

*The reviewer is correct in the interpretation of this correlation, which is surprisingly going in the opposite direction as compared to what we were expecting. We think that children that showed the smaller change in alphabet knowledge are actually those kids that had higher alphabet knowledge at the pre-training session. Hence, they did not have a lot of margin for improvement. What we are observing in this correlation might just be due to a ceiling effect in the alphabet knowledge scale. We updated the paragraph on page 24 to make this point clearer. We do not think that Figure S3 will help better understand the correlations in Table 5 as it does not include information about individual behavioral scores. Hence, we decided to leave it in the Supplementary materials.*

**Reviewer #2:**

The authors have made a commendable effort in addressing the feedback provided by the reviewers. I have minor feedback regarding the description of the behavioral measures: instead of combining everything into one paragraph, I suggest listing the measures separately for greater clarity and readability.

*We changed the format of the paragraph on pages 12-14 following this suggestion.*
